# Chronic Diseases

**Published:** 1887-12

**Authors:** J. G. Gundlach

**Affiliations:** Terrell, Tex.


### CHRONIC DISEASES.

MR. EDITOR: THE HOMŒOPATHIC PHYSICIAN for August just to hand. I was much interested in reading your report of the discussion on the suppression of gonorrhœa at the I. H. A. meeting. It is mighty good reading, as Horace Greeley would say, and it was quite a treat, I can assure you; and the publication of these discussions is a very good idea, as it gives the less fortunate ones an opportunity of being benefited by them, which otherwise they could not unless able to attend the meetings in person. In reading this discussion, I was reminded of the case I herewith send you, which may be of interest to your many readers, and which will further illustrate the truth or fact that in prescribing the homœopathic simillimum in chronic diseases we often bring back the original trouble, whether it be gonorrhœa, measles, or any other disease.

Some three years ago, while in Dr. J. T. Kent's office, Dr. Knot, of Monticello, Ill., related a case of a little girl who had been sick some two years before with an attack of measles, from which she never fully recovered. Just what the condition was at that time I do not remember, but Dr. Knot gave her Carbo-veg. in order to develop his case. (See "The Undeveloped Case," by J. T. K., H. P., January, 1885, p. 25.) In a few days the child broke out with a measley eruption, which ran its course, and the child was again restored to its usual health. My own case is very much like the above, and is as follows:

February 10th, 1887.—Y. A., bright, aged seventeen, was brought to me by his father to get something for his cough. The father gave the following account of the boy's case: About a year and a half ago the boy had an attack of measles, leaving him in delicate health and with a bad cough, which has been going from bad to worse ever since. He is aggravated from the slightest cold he takes, which he seems to take very easily at this time, and for the last month or two, it being winter, he was coughing more or less all the time, worse from changing from warm to cold, or going out-doors, or from a warm room to a cold one. The cough caused a pain in both sides of the chest. Expectoration was greenish-yellow and of a sweet taste. His breath was short, and any extra exertion would bring on a coughing spell. He was weak and much emaciated; face flushed, and had some fever with sweats at night, mostly toward morning. Had no appetite, and did not digest his food. This was about all I could find out. Family history was good. Dr. Knot's case came to my mind, and with the symptoms I had I decided to give him Carbo-veg., feeling sure I should accomplish some result. He got Carbo-veg.$^{8c}$, a powder night and morning for four days, and plenty of Sac. lac., as he lived in the country, with the instructions not to take any other medicine of any kind. Three weeks after the above the father came in town to see me, as he was out of medicine. He told me the boy, after taking the medicine a few days, began to complain more and went to bed with high fever, soon breaking out with first-rate measley eruptions. The boy was sick in bed a week, during which time he got only the Sac. lac. powders. As the eruption was out all right, the father thought it was all he needed, when he began to improve, and was getting better fast, just as fast as he could all the time. A week or two after this the young man came himself, looking well. I hardly knew him. He said he was getting better all the time. Appetite was good, no more fever or sweat, no pain on coughing, of which he only has a slight spell in the morning and in the evening, caused by a tickling under the sternum. I gave him another lot of Sac. lac. and told him should he get worse again at any time to come and I would give him the medicine, as his father, like many more in this country at this time, has not the money to pay, and so had neglected his boy so long. He must be all right, as he never came back. And thus has the truth again been verified. J. G. GUNDLACH, M. D.

TERRELL, TEX.

